# Atypical Presentation of Retrograde Cricopharyngeus Dysfunction in a Patient With Gilles de la Tourette Syndrome

**DOI:** 10.7759/cureus.107406

**Published:** 2026-04-20

**Authors:** Ethan J Jenkins, Solomon Pudi

**Affiliations:** 1 Acute Medicine, Royal Preston Hospital, Manchester, GBR; 2 Otolaryngology, Tameside General Hospital, Manchester, GBR

**Keywords:** botox, botulinum toxin, gilles de la tourette syndrome, r-cpd, retrograde cricopharyngeal dysfunction, retrograde cricopharyngeus dysfunction, tourette syndrome

## Abstract

Retrograde cricopharyngeus dysfunction (R-CPD) is a newly classified syndrome in which a person is unable to burp, submitting them to socially debilitating symptoms of abdominal distension, gurgling noises from the throat and excessive flatulence. We discuss the case of a man in his 30s with Gilles de la Tourette syndrome, who acquires R-CPD following a period of transient dysphagia. The patient experienced years of inconclusive investigations and failed treatments before discovering the condition online and proposing it to his ENT specialist. He was treated with a botulinum toxin (BT) injection into the cricopharyngeus muscle, which showed an initial positive benefit. This case report emphasises the importance of considering R-CPD in patients presenting with similar symptoms. We discuss the diagnostic and therapeutic uses of BT and the additional challenges of a concurrent diagnosis of Tourette syndrome. The awareness of R-CPD amongst medical practitioners is vital to ensure its timely diagnosis, ultimately preventing unnecessary treatments and patient distress.

## Introduction

Retrograde cricopharyngeus dysfunction (R-CPD) is a recently identified syndrome characterised by a set of symptoms stemming from the inability to burp. These symptoms include severe abdominal bloating, gurgling noises from the lower neck, excessive flatulence and, often, difficulty vomiting [1,2]. The condition is believed to be established during childhood [3] and is a result of impaired relaxation of the upper oesophageal sphincter (UOS), specifically the cricopharyngeus muscle (CPM), in response to increased pressure in the oesophagus [4,5]. While the function of the UOS during normal (antegrade) swallowing has been comprehensively studied in conditions such as cricopharyngeal spasm, attention to its retrograde function, which facilitates belching and vomiting, has only recently been highlighted.

The current mainstay treatment for R-CPD is an injection of botulinum toxin (BT) into the CPM to cause muscle relaxation. BT has proven to be highly effective for R-CPD, so much so that a positive response to treatment is now a supportive diagnostic tool [1]. Furthermore, after a single BT injection, many patients experience sustained therapeutic relief well beyond the expected pharmacological effects of BT, which could suggest its transient effect facilitates the retraining of the burp reflex [6].

The recent codification of R-CPD by Bastian and Smithson [1] and its increased awareness, through further published case series, has instigated an upsurge in its research and diagnosis. However, R-CPD still remains a relatively unknown and underecognised medical condition amongst clinicians.

## Case presentation

A man in his 30s presented to the ENT outpatient clinic with the primary complaint of not being able to burp. As a consequence, he suffered from recurrent, painful abdominal distention that could not be relieved. Further symptoms included excessive flatulence and gurgling noises from the chest, and there were no examination findings.

On questioning, the onset of these issues occurred four years prior and coincided with a sudden difficulty in swallowing. This acute dysphagia had no apparent trigger and led to frequent regurgitation of both solids and liquids, along with the sensation of boluses becoming stuck retrosternally. Over the course of three months, the patient’s swallowing function gradually returned to normal. During this period, however, he experienced significant weight loss due to poor oral intake and underwent a series of inconclusive investigations and treatments. Despite this improvement, his symptoms of abdominal distention and inability to burp persisted.

In his early childhood, he was diagnosed with the neurodevelopmental disorder, Gilles de la Tourette syndrome. While his condition had improved into adulthood, he struggled with everyday premonitory urges to effectuate ticks such as teeth chomping, grunting, blinking, abdomen tensing and vocal outbursts. He currently takes no medications and instead manages his Tourette’s syndrome with self-taught cognitive behavioural therapy techniques. This included the focused redirection of sensations into a different part of his body, such as his feet. The patient had no significant family history. He did not smoke and had reduced his alcohol intake to the occasional drink since it worsened his abdominal symptoms. Further areas of his life affected include cessation of hobbies such as weight-lifting and mixed martial arts, as well as adapting his work from manual labour to office-based.

Investigations and differential diagnoses

Due to the initial presentation of dysphagia, the patient was referred to the two-week wait cancer pathway for urgent gastroscopy. The endoscopy was reported as unremarkable with no evidence of physical obstruction, such as stricture or mass. At a gastroenterology follow-up, he reported having no success with pharmaceutical treatment such as omeprazole, which brought out a rash, and ranitidine, which had no effect. Next, an oesophageal manometry and 24hr pH/impedance study was requested for suspicion of achalasia and other motility disorders, for which both proved to be normal. A gastroenterologist postulated that these were the results of someone with a reflux hypersensitivity, typically treated with oesophageal neuromodulators such as tricyclic antidepressants. Treatment was declined by the patient due to concerns of the medication affecting his Tourette’s syndrome, and a second opinion was sought from ENT.

**Figure 1:**
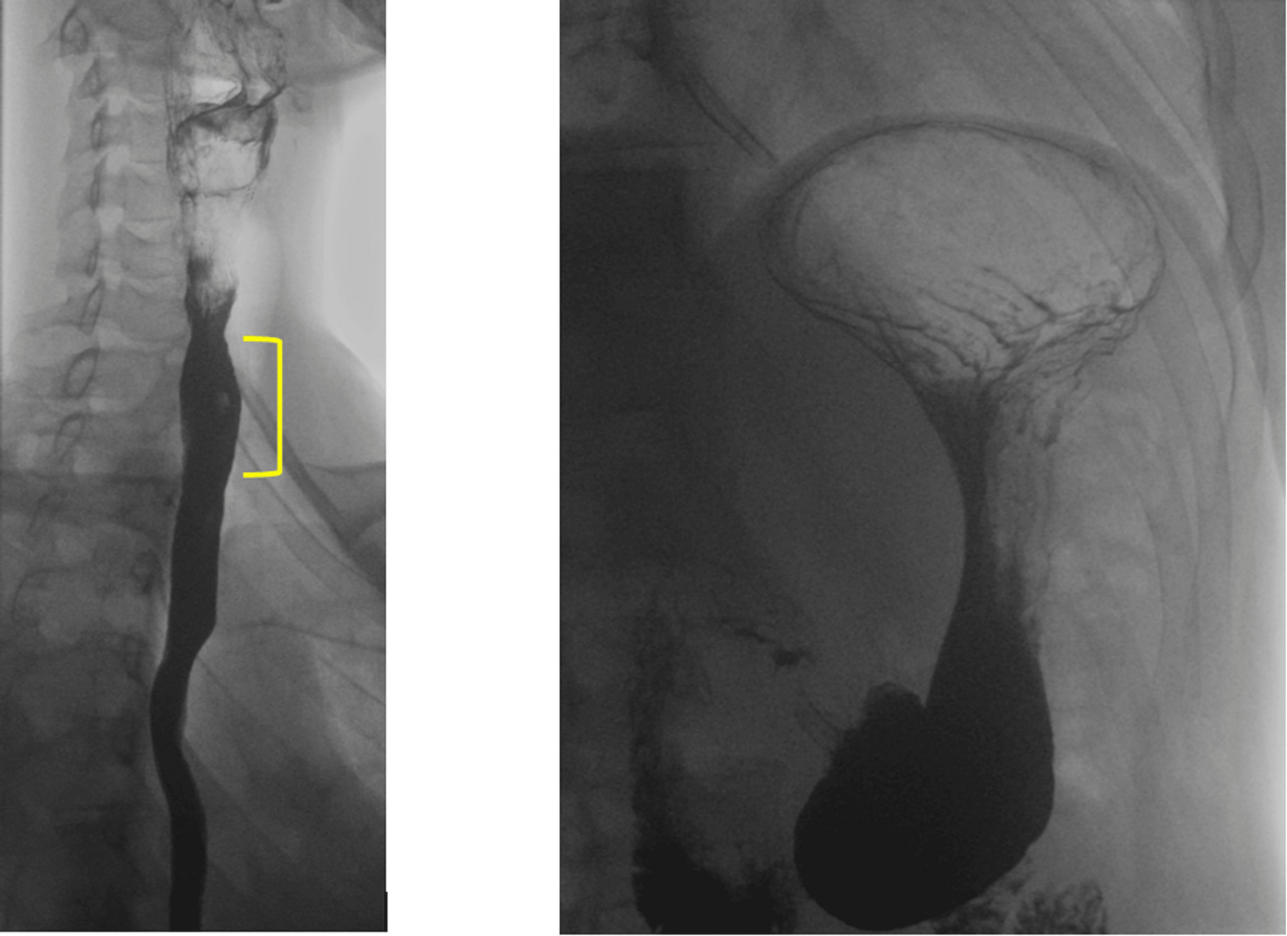
Barium swallow images showing gaseous distention of the upper oesophagus (left) and the stomach (right)

The patient later presented to his ENT appointment with the self-proposed diagnosis of R-CPD after having come across it online. Following a normal examination with flexible nasoendoscopy (FNE) and normal barium swallow, the consultant agreed that the patient’s clinical symptoms indeed matched the hypothesised diagnosis. On review of the investigations, with the diagnosis in hindsight, the barium swallow in fact reveals gaseous distention of the stomach and oesophagus (Figure 1), seen similarly in other R-CPD case reports [7]. Barium swallow, pH measurement, and manometry have so far proved inconsistent in R-CPD diagnosis and therefore should only be done with the aim of excluding other conditions such as achalasia. Instead, the condition is diagnosed through the history and clinical examination using FNE, and its subsequent response to treatment.

Treatment

Since the patient’s symptoms were congruent to those of R-CPD, it was decided to carry out an injection of BT into the CPM for diagnostic certainty, and ultimately to provide symptomatic relief. The patient was counselled on the potential post-operative effects of transient alteration in swallowing and worsening of existing reflux. Under general anaesthetic, a Weerda diverticuloscope was advanced into the hypopharynx to aid visualisation of the UOS and a total of 10 units of BT (0.1mL of a 100 units/mL saline solution) was administered into the CPM via multiple injection sites. There were no anaesthetic or surgical complications.

Outcome

At six weeks follow-up, the patient reported a return in the ability to burp and no longer suffered from debilitating abdominal symptoms. However, as speculated, he had begun experiencing worsening reflux. This was predominantly in the form of prolonged episodes of heartburn, particularly at night when lying flat. Unfortunately, by the next three-month follow-up, he indicated that his condition had returned to baseline after roughly six weeks of therapeutic benefit. A second injection of BT was offered but declined, as the patient wished to continue exploring conservative measures such as dietary and lifestyle modification.

## Discussion

The term R-CPD was first coined in a case series by Bastian and Smithson in 2019 [1]. After successfully reversing a patient’s inability to belch with an injection of BT, Bastian was astonished to find emails from similar patients self-presenting to him. Unbeknownst to him, the initial patient had shared his experience on the forum website, Reddit, where it had been read and sympathised with by many desperate, undiagnosed patients, all with similar stories of debilitating symptoms, self-imposed lifestyle restrictions and avoidance of social activities. Likewise, in this case report, a common theme in R-CPD case literature is patients self-present already with the diagnosis in mind, suggesting deficient awareness amongst physicians, but also the increasing application of the internet in medicine.

Since the condition was first described, further case series have been released, providing similar insights into R-CPD’s symptoms and the efficacy of BT injections [6-9]. A notable commonality among R-CPD patients is their consistent recollection of having had the condition for as long as they can remember, with only one of the 51 patients in Bastian's initial study able to place when their symptoms first developed [1]. Building on this, a study of paediatric R-CPD patients suggests the condition typically emerges in childhood, making it atypical for our patient to have developed R-CPD in his 30s [3]. Furthermore, there have been no published cases of "acquired" R-CPD that have occurred spontaneously alongside dysphagia, despite research raising questions about the role of functional dysphagia in propagating R-CPD symptoms [2].

**Figure 2:**
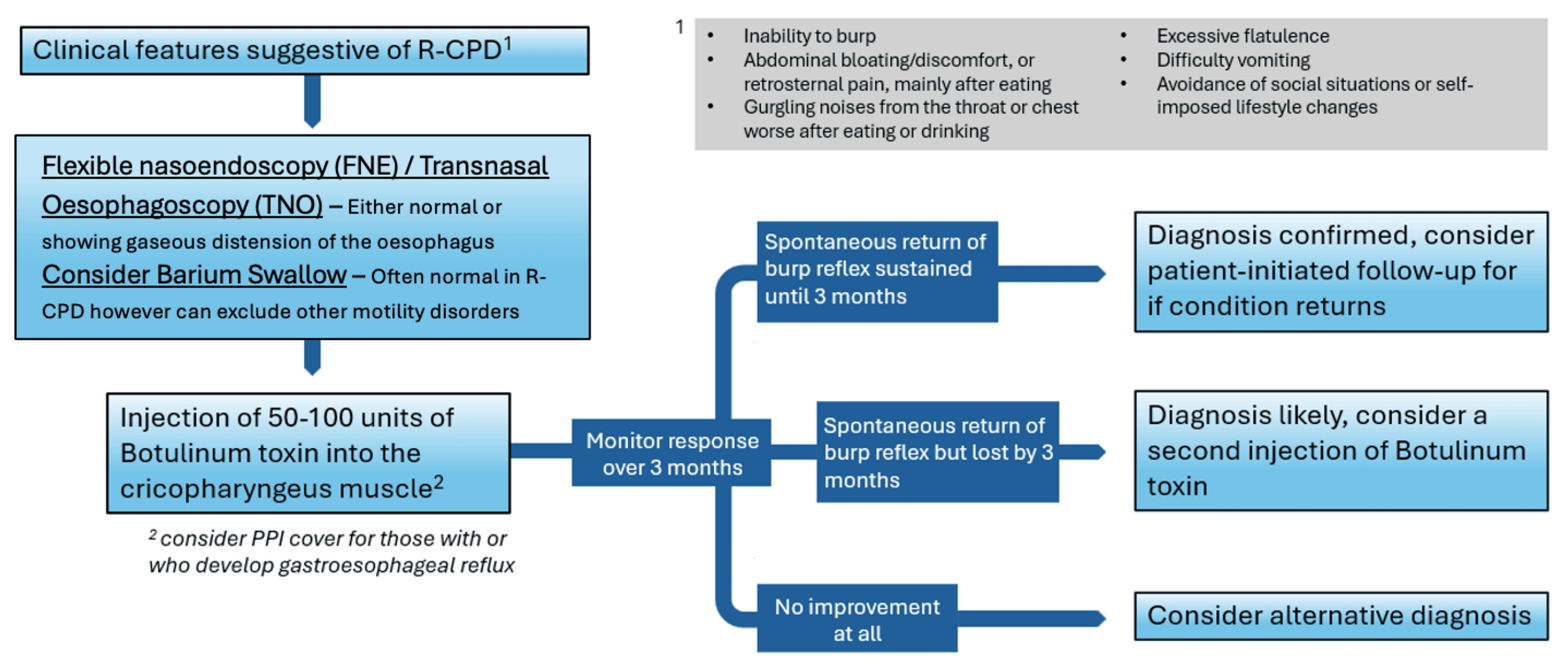
A proposed management pathway for suspected R-CPD based on wider literature R-CPD: retrograde cricopharyngeus dysfunction; PPI: proton pump inhibitor

Given that R-CPD is a relatively novel diagnosis, there are no official guidelines on its diagnosis and management. In Figure 2, we present a proposed management algorithm, based on published research, which can be implemented once R-CPD has been clinically suspected.

Questions also remain about optimal BT dosing regimens, as there are currently no large prospective trials determining the dose of BT needed to successfully treat R-CPD. This has led to a wide range of trialled doses, from 10U to 100U [7,9]. One retrospective case series shows that a single dose of 50U BT is 99.5% effective, with 79.9% of patients maintaining the ability to burp for up to six months [6]. Even lower recurrence rates, without an associated increase in adverse effects, have been shown with higher doses like 100U [7], suggesting that 10U may have been a suboptimal dose in this patient report. Not only that, in the same study, those who didn't establish long-lasting therapeutic benefit did so instead after a second BT injection, demonstrating that a further dose may have been beneficial for this patient. Alternatively, those with Tourette’s syndrome and other neurological disorders may face poorer outcomes to BT injections, since it has been postulated that re-establishing afferent-efferent feedback to the brain is crucial for the treatment’s lasting effect [1].

Finally, the potential interplay of the patient’s concurrent diagnosis of Gilles de la Tourette syndrome is also of interest. The neurodevelopmental disorder is most affiliated with motor and vocal tics; however, patients also experience troublesome sensory phenomena [10]. These are in the form of premonitory urges, which are relinquished by the tic itself, or sensory hypersensitivity, the constant exaggerated awareness of external or internal stimuli [11]. These can manifest themselves in a myriad of forms, and it wouldn’t be implausible to suggest that the sensation of needing to belch is a sensory phenomenon or at least amplified stimuli. Conceivably, this association remains speculative, and the presence of Tourette’s syndrome may represent a coincidental comorbidity rather than a direct contributing factor to symptoms consistent with R-CPD.

For this reason, management should primarily follow established approaches for R-CPD. Considering the patient’s symptoms within the framework of Tourette’s syndrome may only warrant further exploration if they prove refractory to standard treatment. Nevertheless, effective control of Tourette’s symptoms may still play a supportive role in improving tolerance of organic sensory stimuli [12]. One last aspect in which Tourette’s could aggravate the experience of R-CPD is through covert air swallowing when effectuating or resisting tics. Aerophagia has, on occasion, been linked with the condition, and undoubtedly the accumulation of additional air into the stomach would only compound the experienced abdominal distention and discomfort [13,14].

## Conclusions

In summary, this case describes a patient in his 30s with Gilles de la Tourette syndrome, who acquires R-CPD following a transient period of acute dysphagia. We discuss how limitations in health practitioner awareness mean patients with R-CPD often endure years of distress while undergoing unnecessary and inconclusive tests and treatments. With adequate clinical knowledge, the condition can be diagnosed and treated effectively, often with a single dose of BT, thus mitigating patient anguish and strain on other healthcare resources. R-CPD should therefore remain a top differential in all patients presenting with issues stemming from difficulty burping.

The case also explores the additional considerations in patients with coexisting Tourette syndrome, who may be at risk of having their symptoms overlooked or misattributed. While no causal relationship between the two conditions is suggested, this report offers insight into the complexities of managing overlapping presentations and underscores the importance of a thoughtful, individualised approach to care.

## References

[1] Bastian RW, Smithson ML (2019). Inability to belch and associated symptoms due to retrograde cricopharyngeus dysfunction: diagnosis and treatment. OTO Open.

[2] Miller ME, Lina I, Akst LM (2024). Retrograde cricopharyngeal dysfunction: a review. J Clin Med.

[3] Hoffman MR, Schiffer B, Patel RA, Smith ME (2022). "I've never been able to burp": preliminary description of retrograde cricopharyngeal dysfunction in children. Int J Pediatr Otorhinolaryngol.

[4] Oude Nijhuis RA, Snelleman JA, Oors JM (2022). The inability to belch syndrome: a study using concurrent high-resolution manometry and impedance monitoring. Neurogastroenterol Motil.

[5] Kahrilas PJ (2022). Retrograde upper esophageal sphincter function… and dysfunction. Neurogastroenterol Motil.

[6] Hoesli RC, Wingo ML, Bastian RW (2020). The long-term efficacy of botulinum toxin injection to treat retrograde cricopharyngeus dysfunction. OTO Open.

[7] Karagama Y (2021). Abelchia: inability to belch/burp-a new disorder? Retrograde cricopharyngeal dysfunction (RCPD). Eur Arch Otorhinolaryngol.

[8] Wajsberg B, Hoesli RC, Wingo ML, Richardson BE, Bastian RW (2022). Retrograde cricopharyngeus dysfunction: an orphan disease?. Am J Gastroenterol.

[9] Pavesi L, Balzano C, Mauramati S (2023). Retrograde cricopharyngeus dysfunction effectively treated with low dose botulinum toxin. A case report from Italy. Front Neurol.

[10] Cohen AJ, Leckman JF (1992). Sensory phenomena associated with Gilles de la Tourette's syndrome. J Clin Psychiatry.

[11] Isaacs D, Riordan H (2020). Sensory hypersensitivity in Tourette syndrome: a review. Brain Dev.

[12] Bliss J (1980). Sensory experiences of Gilles de la Tourette syndrome. Arch Gen Psychiatry.

[13] Frye RE, Hait EJ (2006). Air swallowing caused recurrent ileus in Tourette's syndrome. Pediatrics.

[14] Weil RS, Cavanna AE, Willoughby JM, Robertson MM (2008). Air swallowing as a tic. J Psychosom Res.

